# Contemporary Trends in Disparities for Renal Artery Stenting and Angioplasty in the United States

**DOI:** 10.1016/j.jscai.2025.102610

**Published:** 2025-09-30

**Authors:** Waseem Wahood, Edwin A. Takahashi, Andrew H. Stockland, Christopher J. Reisenauer, Lilach O. Lerman, Sanjay Misra

**Affiliations:** a Department of Interventional Radiology, University of Miami Miller School of Medicine/Jackson Memorial Hospital, Miami, Florida; b Department of Radiology, Division of Vascular and Interventional Radiology, Mayo Clinic, Rochester, Minnesota; c Division of Nephrology and Hypertension, Mayo Clinic, Rochester, Minnesota

**Keywords:** endovascular intervention, racial disparity, renal artery stenosis, trends

## Abstract

**Background:**

Over the past 2 decades, several randomized clinical studies, including the STAR, ASTRAL, and CORAL trials, investigated interventions for atherosclerotic renal artery stenosis (RAS). However, a gap exists in the available data concerning racial disparities in the context of endovascular intervention for RAS. To address this gap, we conducted a comprehensive investigation utilizing a national database.

**Methods:**

The National Inpatient Sample was queried between 2005 to 2019, for adult patients with RAS. Endovascular interventions included angioplasty and/or stenting. The Cochran-Armitage test was conducted to assess trends in the proportion of endovascular intervention among races. Multivariable logistic regression was used to assess the patient profile of those who received endovascular intervention, nonroutine discharge, and in-hospital mortality. Admissions involving fibromuscular dysplasia and open intervention were excluded.

**Results:**

In total, 792,108 admissions involving RAS were identified; 634,801 were White patients, 80,585 were Black patients, 44,415 were Hispanic, 12,409 were Asian or Pacific Islander, 3879 were Native American, and 16,019 were of other race. The proportion of utilization of endovascular procedures was trending downward by an average of –2.0% per year (*P* < .001). The proportion of White patients who underwent renal stent placement decreased by –2.0% per year, Black patients decreased by –1.5% per year, Hispanic patients decreased by –2.2% per year, Asian or Pacific Islander patients decreased by –1.9% per year, Native American patients decreased by –2.4% per year, and those of the other race decreased by –1.8% per year (all *P* < .001). Compared to White patients, Black patients had lower odds of undergoing endovascular procedure (OR, 0.80; *P* < .001), lower odds of routine discharge (OR, 0.86; *P* < .001), and lower odds of in-hospital mortality (OR, 0.86; *P* = .023).

**Conclusions:**

Over the 15-year study period, there was a decreasing trend in the proportion of endovascular intervention for RAS for both White and non-White patients, though the decrease in trend was larger among White patients. Additionally, Black patients had lower odds of undergoing endovascular treatment, lower odds of routine discharge, and lower odds of mortality compared to White patients.

## Introduction

In the 2017 guidelines from the Society for Cardiovascular Angiography & Interventions (SCAI), renal artery stenting was advised for hemodynamically significant atherosclerotic renal artery stenosis (RAS), considering anatomic and clinical scenarios.^1^ This was in agreement with the Kidney Disease: Improving Global Outcomes (KDIGO) Controversies Conference in 2020.^2^ Meanwhile, the European guidelines from 2017 state that angioplasty may be considered in patients with RAS in addition to significant cardiovascular diseases.^3^ Although there exists some discordance among these guidelines, the 3 major trials conducted within the last 2 decades all concluded that revascularization of the renal artery plus optimal medical therapy was not any more beneficial than medical therapy alone for RAS.^4^, ^5^, ^6^ Although the trials and guidelines have shaped the patterns of endovascular treatment for RAS, they fall short in addressing the racial and ethnic considerations associated with treating this specific disease process. A significant gap persists in our understanding, as there are limited data on racial disparities in the context of renal artery stenting for RAS. To bridge this knowledge gap, we undertook an investigation utilizing a national database.

## Methods

### Data source

The National Inpatient Sample (NIS) database was queried from January 1, 2005 to December 31, 2019. This database samples 7 million hospitalizations per year representing 20% of all discharges annually and is created by the Healthcare Cost and Utilization Project. This database is managed by the Agency for Healthcare Research and Quality and is the largest public national all-payer database.^7^ Furthermore, the NIS contains weighted discharge information that allows investigators to provide national estimates. IRB approval was not required for this study; all included data were deidentified.

### Cohort selection

The International Classification of Diseases, 9th and 10th editions (ICD-9 and ICD-10), were used to identify hospitalizations involving adult patients with RAS. Endovascular interventions included angioplasty and/or stenting. Admissions involving fibromuscular dysplasia, mesenteric ischemia, vascular trauma, and open intervention were excluded to mitigate bias in treatment and discharge disposition. ICD codes utilized in this study can be seen in Supplemental Table S1.

### Outcomes and variables of interest

The following demographic variables were extracted: age, race (White, Black, Hispanic, Asian American and Pacific Islander [AAPI], Native American, and other), gender, insurance type, Elixhauser comorbidity indices (ECI), and CHA_2_DS_2_-VASc score. NIS classifies Hispanic as “race,” such that Hispanic ethnicity takes precedence over race when a patient is classified as Hispanic and a certain race.^7^ This method was similar to other studies analyzing racial and ethnic disparities among different disease processes and their treatments.^8^, ^9^, ^10^, ^11^, ^12^, ^13^

Elixhauser comorbidity indices are based on 31 predefined comorbidities that utilize ICD-9 and ICD-10 codes to identify the risk of poor outcomes.^14^ A summative score is created with the weighting of each comorbidity ranging from –7 to +12. Lower numbers indicate a lower risk, with the lowest possible being –19 and the highest possible being +89. The CHA_2_DS_2_-VASc score is an established prediction that estimates the baseline risk of a thromboembolic event in cardiac patients.^15^ This score was calculated using variables available from the ECI and Charlson comorbidity index in addition to other variables required to calculate the score using ICD-9 and ICD-10 codes. This method was previously used and validated; accurate definitions of each component were obtained from guidelines and surveys published by the European Society of Cardiology, American Heart Association, and American College of Cardiology.^16^, ^17^, ^18^, ^19^ The following hospital data were collected: bed size, location (rural/urban), teaching status, and region (Northeast, Midwest, South, and West). Discharge disposition was divided into routine (discharge to home or self-care) and nonroutine discharge (transfer to short-term hospital, transfer to skilled nursing facility or intermediate care facility, home health care). The outcome analyzed was “routine discharge.”

### Statistical analysis

Continuous variables are reported as mean and standard error (SE). Categorical data are reported as frequencies and percentages. χ^2^ tests were used to compare the proportions of each categorical variable between the 6 groups. ANOVA or Kruskal-Wallis tests were used to compare the means of continuous variables between 6 groups of normally or nonnormally distributed data, respectively.

The Cochran-Armitage test was conducted to test for a linear trend in the proportions of utilization of endovascular therapy for RAS. The numerators were the number of interventions among each race and the denominator was the total number of admissions involving each race with RAS. Trend lines were compared between the 6 race groups using linear regression analysis, which produces a mean difference and 95% CI in trend compared to the reference group, White race.^20^ Additionally, trend lines were plotted for average ECI score and CHA_2_DS_2_-VASc score per year by procedure and by race, regardless of whether they received the intervention, to assess average illness severity per year.

Hierarchical multivariable logistic regression analysis, stratified by NIS-defined strata and year, was conducted to assess patient profiles for endovascular intervention, routine discharge, and in-hospital mortality. Results were presented as odds ratios (OR) and their respective 95% CI. To further explore the interaction between race and endovascular intervention on the odds of routine discharge, we conducted a marginal effect analysis. This analysis quantifies the difference in the predicted probability of endovascular intervention on the likelihood of routine discharge across the race/ethnicity groups.^21^ These results were presented as percent change in the predicted probability of routine discharge among each race and comparing the change in predicted probability among each race to that of the White patients. Marginal effect analysis was also conducted for the interaction between race and several comorbidities, including obesity, CKD stage ≥3, and complicated diabetes on the odds of receiving endovascular intervention. *P* values <.05 were considered statistically significant. All estimates were nationalized using discharge weighting provided by the Healthcare Cost and Utilization Project. Statistical analysis was performed using STATA 17 (StataCorp LLC).

## Results

### Patient demographics

In total, there were 792,108 admissions over the 16-year period. Among these admissions, 634,801 (80.1%) were White, 80,585 (10.2%) were Black, 44,415 (5.6%) were Hispanic, 12,409 (1.6%) were AAPI, 3879 (0.49%) were Native American, and 16,019 (2.0%) were classified as other race. The average age for White admissions was 73.6 ± 0.06 years, 67.0 ± 0.15 years for Black patients, 69.6 ± 0.21 years for Hispanic patients, 71.9 ± 0.33 for AAPI patients, 69.8 ± 0.64 for Native American patients, and 70.7 ± 0.27 years for other race. Per race, women comprised 58% of White patients, 68% of Black patients, 60% of Hispanic patients, 56% of AAPI patients, 61% of Native American patients, and 57% of the other race. Additional characteristics are summarized in Table 1.

**Table 1:** Patient demographics by race.

Variables	White (n = 634,801)	Black (n = 80,585)	Hispanic (n = 44,415)	Asian/Pacific Islander (n = 12,409)	Native American (n = 3879)	Other (n = 16,019)
Age, y	73.63 (0.06)	67.03 (0.15)	69.59 (0.21)	71.93 (0.33)	69.79 (0.64)	70.70 (0.27)
Length of stay, dd	5.15 (0.03)	6.11 (0.07)	5.75 (0.10)	6.06 (0.14)	4.90 (0.25)	5.75 (0.15)
Elixhauser scaled mortality index	12.00 (0.07)	12.38 (0.10)	11.19 (0.17)	12.92 (0.21)	11.04 (0.42)	11.45 (0.22)
Total charge in 2020 dollars	17,309.06 (108.79)	17,468.60 (194.50)	19,835.11 (336.60)	23,005.52 (591.71)	18,194.54 (754.65)	20,547.70 (505.65)
CHA2DS2-VASc score	4.58 (0.01)	4.41 (0.02)	4.51 (0.02)	4.70 (0.03)	4.38 (0.06)	4.48 (0.03)
Female patients	367,550 (57.90%)	54,698 (67.89%)	26,595 (59.89%)	6988 (56.32%)	2360 (60.85%)	9116 (56.91%)
Endovascular procedure (angioplasty and/or stenting)	103,970 (16.38%)	10,588 (13.14%)	8110 (18.26%)	1871 (15.08%)	751 (19.37%)	2731 (17.05%)
Angioplasty	96,771 (15.24%)	9810 (12.17%)	7590 (17.09%)	1702 (13.72%)	686 (17.70%)	2511 (15.68%)
Stenting	80,180 (12.63%)	7485 (9.29%)	5720 (12.88%)	1400 (11.28%)	567 (14.63%)	2123 (13.25%)
Long-term aspirin	97,613 (15.38%)	11,463 (14.23%)	5520 (12.43%)	1779 (14.34%)	624 (16.09%)	2014 (12.57%)
Long-term anticoagulant	50,550 (7.96%)	4254 (5.28%)	2429 (5.47%)	715 (5.76%)	138 (3.56%)	965 (6.02%)
Long-term antiplatelet/antithrombotics	42,166 (6.64%)	4855 (6.02%)	2781 (6.26%)	685 (5.52%)	276 (7.11%)	974 (6.08%)
Other aortic procedures	5873 (0.93%)	506 (0.63%)	221 (0.50%)	130 (1.05%)	39 (1.01%)	147 (0.92%)
EVAR	11,302 (1.78%)	466 (0.58%)	447 (1.01%)	210 (1.70%)	73 (1.89%)	205 (1.28%)
CKD stage 3+	166,110 (26.17%)	27,246 (33.81%)	12,887 (29.02%)	4511 (36.35%)	1001 (25.81%)	4182 (26.11%)
Region of hospital						
Northeast	114,943 (18.11%)	12,520 (15.54%)	7349 (16.55%)	2075 (16.73%)	316 (8.13%)	4051 (25.29%)
Midwest	159,394 (25.11%)	16,612 (20.61%)	2972 (6.69%)	1285 (10.35%)	660 (17.02%)	2855 (17.82%)
South	271,892 (42.83%)	44,581 (55.32%)	19,610 (44.15%)	2426 (19.55%)	2194 (56.58%)	6546 (40.87%)
West	88,572 (13.95%)	6872 (8.53%)	14,484 (32.61%)	6622 (53.37%)	709 (18.27%)	2566 (16.02%)
Bed size of hospital (strata)						
Small	84,901 (13.42%)	9101 (11.34%)	5313 (11.99%)	1455 (11.76%)	820 (21.27%)	2374 (14.91%)
Medium	160,364 (25.34%)	20,679 (25.78%)	10,866 (24.52%)	2973 (24.03%)	1060 (27.51%)	3676 (23.09%)
Large	387,556 (61.24%)	50,442 (62.88%)	28,128 (63.49%)	7946 (64.21%)	1973 (51.22%)	9872 (62.00%)
Median household income national quartile for patient ZIP code						
0-25 percentile	160,501 (25.71%)	42,183 (53.45%)	17,456 (40.31%)	1753 (14.34%)	1649 (44.73%)	4253 (27.39%)
26-50 percentile	177,663 (28.46%)	16,576 (21.00%)	10,131 (23.39%)	2113 (17.28%)	1066 (28.91%)	3647 (23.49%)
51-75 percentile	156,519 (25.08%)	12,238 (15.51%)	9165 (21.16%)	3290 (26.90%)	574 (15.56%)	4013 (25.85%)
>75 percentile	129,503 (20.75%)	7928 (10.04%)	6556 (15.14%)	5071 (41.48%)	398 (10.80%)	3611 (23.26%)
Location/teaching status of hospital (strata)						
Rural	67,065 (10.60%)	4623 (5.76%)	1933 (4.36%)	403 (3.26%)	863 (22.41%)	986 (6.19%)
Urban nonteaching	251,267 (39.71%)	21,711 (27.06%)	17,591 (39.70%)	4641 (37.51%)	1335 (34.66%)	6266 (39.35%)
Urban teaching	314,489 (49.70%)	53,888 (67.17%)	24,782 (55.93%)	7330 (59.24%)	1654 (42.93%)	8670 (54.46%)
Routine discharge	383,802 (61.84%)	49,606 (62.68%)	29,253 (67.11%)	7680 (64.01%)	2677 (71.05%)	10,056 (64.36%)
Died during hospitalization	13,791 (2.17%)	1408 (1.75%)	774 (1.75%)	391 (3.15%)	110 (2.84%)	381 (2.38%)
Congestive heart failure	235,149 (37.04%)	34,497 (42.81%)	16,226 (36.53%)	4762 (38.38%)	1412 (36.41%)	5776 (36.05%)
Cardiac arrhythmias	233,659 (36.81%)	21,653 (26.87%)	12,292 (27.68%)	3987 (32.13%)	1188 (30.63%)	4941 (30.85%)
Valvular disease	112,893 (17.78%)	11,957 (14.84%)	6291 (14.16%)	1909 (15.38%)	600 (15.48%)	2556 (15.96%)
Pulmonary circulation disorders	52,520 (8.27%)	8061 (10.00%)	2855 (6.43%)	880 (7.09%)	264 (6.81%)	1118 (6.98%)
Paralysis	8346 (1.31%)	1477 (1.83%)	625 (1.41%)	291 (2.34%)	44 (1.13%)	242 (1.51%)
Other neurological disorders	44,253 (6.97%)	6978 (8.66%)	2688 (6.05%)	765 (6.17%)	245 (6.32%)	1121 (7.00%)
Chronic pulmonary disease	220,832 (34.79%)	23,998 (29.78%)	9756 (21.97%)	2729 (21.99%)	1278 (32.95%)	4403 (27.49%)
Diabetes, uncomplicated	136,658 (21.53%)	21,788 (27.04%)	13,275 (29.89%)	3216 (25.92%)	1076 (27.74%)	4379 (27.34%)
Diabetes, complicated	77,627 (12.23%)	14,787 (18.35%)	10,002 (22.52%)	2896 (23.34%)	674 (17.39%)	2554 (15.94%)
Hypothyroidism	108,904 (17.16%)	6498 (8.06%)	6080 (13.69%)	1531 (12.34%)	543 (13.99%)	2267 (14.15%)
Renal failure	279,753 (44.07%)	42,680 (52.96%)	20,901 (47.06%)	6855 (55.24%)	1579 (40.72%)	7012 (43.77%)
Liver disease	15,426 (2.43%)	2710 (3.36%)	1530 (3.44%)	421 (3.39%)	86 (2.22%)	426 (2.66%)
Peptic ulcer disease excluding Bleeding	7195 (1.13%)	1035 (1.28%)	441 (0.99%)	204 (1.65%)	42 (1.09%)	196 (1.22%)
AIDS/HIV	407 (0.06%)	323 (0.40%)	80 (0.18%)	<11	<11	<11
Lymphoma	4121 (0.65%)	404 (0.50%)	239 (0.54%)	60 (0.49%)	<11	98 (0.61%)
Metastatic cancer	8517 (1.34%)	943 (1.17%)	330 (0.74%)	132 (1.07%)	25 (0.64%)	158 (0.98%)
Solid tumor without metastasis	22,270 (3.51%)	2466 (3.06%)	1071 (2.41%)	353 (2.84%)	94 (2.43%)	439 (2.74%)
Rheumatoid arthritis/collagen vascular	23,092 (3.64%)	2640 (3.28%)	1502 (3.38%)	390 (3.14%)	126 (3.26%)	513 (3.20%)
Coagulopathy	31,851 (5.02%)	3773 (4.68%)	2040 (4.59%)	918 (7.40%)	113 (2.92%)	894 (5.58%)
Obesity	58,012 (9.14%)	10,736 (13.32%)	5015 (11.29%)	574 (4.63%)	296 (7.64%)	1426 (8.90%)
Weight loss	29,930 (4.71%)	4416 (5.48%)	1822 (4.10%)	645 (5.20%)	175 (4.52%)	821 (5.12%)
Fluid and electrolyte disorders	191,837 (30.22%)	27,471 (34.09%)	14,472 (32.58%)	4765 (38.40%)	1122 (28.94%)	4850 (30.28%)
Blood loss anemia	9407 (1.48%)	1213 (1.51%)	657 (1.48%)	172 (1.38%)	60 (1.53%)	221 (1.38%)
Deficiency anemia	28,565 (4.50%)	4919 (6.10%)	2,259 (5.09%)	614 (4.95%)	159 (4.09%)	876 (5.47%)
Alcohol abuse	13,793 (2.17%)	2307 (2.86%)	827 (1.86%)	92 (0.74%)	54 (1.39%)	269 (1.68%)
Drug abuse	9349 (1.47%)	3832 (4.76%)	680 (1.53%)	104 (0.84%)	89 (2.30%)	211 (1.32%)
Psychoses	5103 (0.80%)	1176 (1.46%)	216 (0.49%)	86 (0.70%)	36 (0.93%)	137 (0.85%)
Depression	69,795 (10.99%)	6337 (7.86%)	4072 (9.17%)	825 (6.65%)	384 (9.89%)	1356 (8.47%)
Hypertension, uncomplicated	253,159 (39.88%)	27,687 (34.36%)	17,033 (38.35%)	4184 (33.72%)	1639 (42.26%)	6410 (40.01%)
Hypertension, complicated	317,886 (50.08%)	48,613 (60.33%)	24,173 (54.43%)	7672 (61.82%)	1886 (48.63%)	8064 (50.34%)
Hypertension, any	567,437 (89.39%)	75,644 (93.87%)	40,868 (92.01%)	11,758 (94.76%)	3502 (90.28%)	14,372 (89.72%)

Values are mean (SE) or n (%).

CKD, chronic kidney disease; EVAR, endovascular aneurysm repair.

### Trend analysis

**Figure 1:**
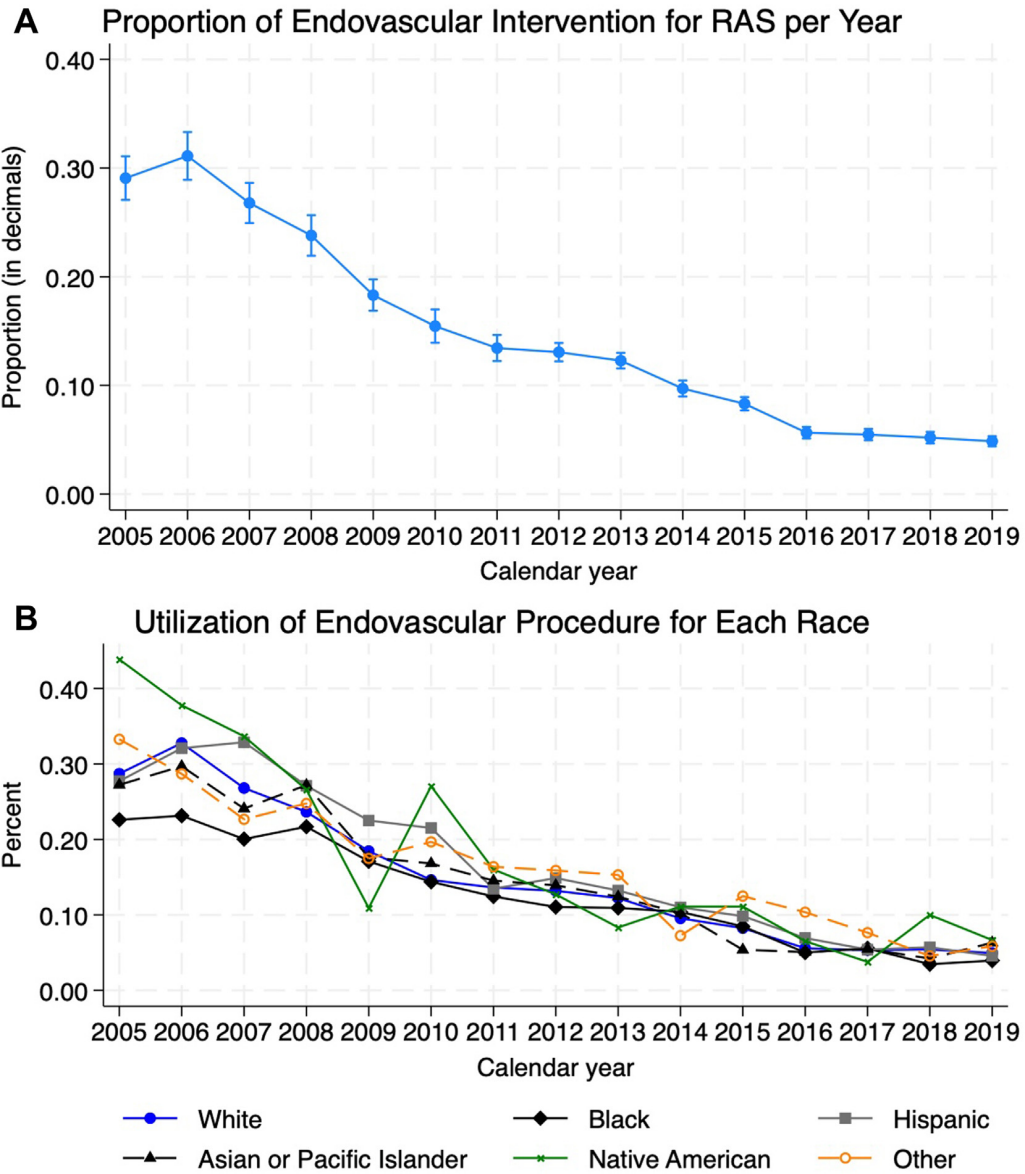
**(A) Proportion of endovascular interventions for renal artery stenosis (RAS) per year, (B) Proportion of utilization of endovascular intervention for each race**.

The proportion of endovascular procedure utilization trended downward by an average of –2.0% per year (95% CI, –2.1 to –1.9; *P* < .001). The proportion of White patients who underwent stenting decreased by –2.0% per year (95% CI, –2.2 to –1.9), Black patients decreased by –1.5% per year (95% CI, –1.7 to –1.3), Hispanic patients decreased by –2.2% per year (95% CI, –2.5 to –1.9), AAPI patients decreased by 1.9% per year (95% CI, –2.3 to –1.6), Native American patients decreased by –2.4% per year (95% CI, –3.6 to –1.3), and those of the other race decreased by –1.8% per year (95% CI, –2.2 to –1.4; all *P* < .001). These trends can be seen in Figure 1A.

Compared to admissions involving White patients, the rate of admissions involving Black patients who received endovascular procedures was lower by 0.52% per year (95% CI, 0.33-0.71; *P* < .001). Admissions involving Hispanic, AAPI, Native American, and other race patients had similar trend lines compared to White patients. These trends can be seen in Figure 1B and Table 2.

**Table 2:** Slopes for the trend in the utilization of endovascular intervention by race and comparison of slopes with White race as the reference group.

	Slope	95% lower bound	95% upper bound	*P* value
Endovascular intervention	–2.00%	–2.14%	–1.86%	<.001
White	–2.04%	–2.19%	–1.90%	<.001
Black	–1.52%	–1.71%	–1.34%	<.001
Hispanic	–2.17%	–2.49%	–1.85%	<.001
Asian	–1.91%	–2.25%	–1.57%	<.001
Native American	–2.44%	–3.60%	–1.28%	<.001
Other	–1.82%	–2.24%	–1.40%	<.001
vs White	base			
Black	0.52%	0.33%	0.71%	<.001
Hispanic	–0.12%	–0.43%	0.18%	.429
AAPI	0.13%	–0.25%	0.52%	.496
Native American	–0.40%	–1.47%	0.68%	.471
Other	0.22%	–0.20%	0.64%	.303

AAPI, American and Pacific Islander.

**Figure 2:**
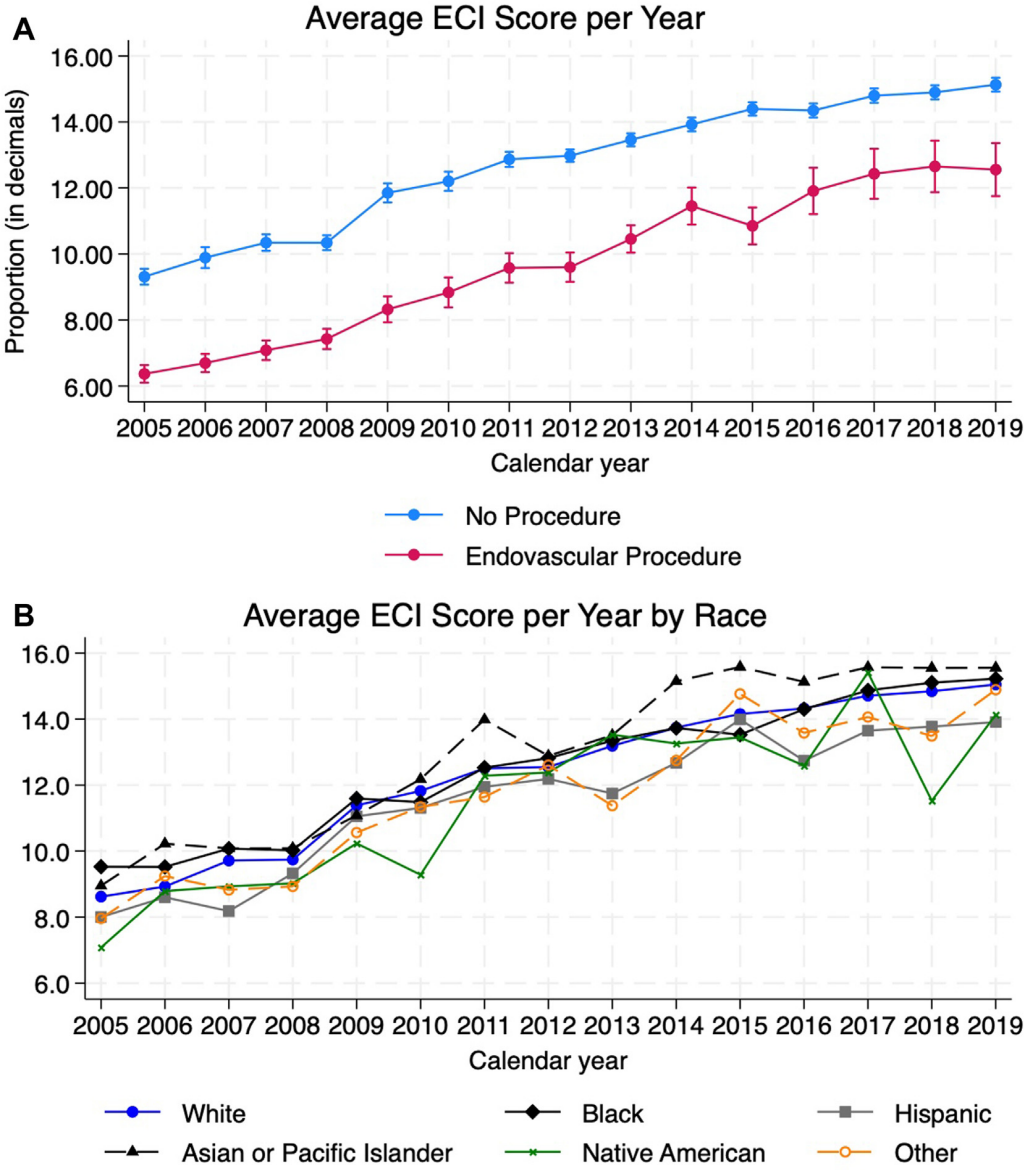
**(A) Average Elixhauser comorbidity index (ECI) score per year, (B) Average ECI score per year by race**.

The Elixhauser comorbidity indices score increased every year by an average of 0.42 per year among those who did not receive an intervention (95% CI, 0.40-0.44; *P* < .001) and 0.49 among those who received an endovascular intervention (95% CI, 0.46-0.53; *P* < .001). This indicates a trend of increasing poor outcome risk. There was an average difference in slope of 0.07 between the 2 groups (95% CI, 0.04-0.11; *P* < .001). Compared to White patients, the average ECI for Black patients was significantly lower by an average of –0.05 (95% CI, –0.08 to –0.01; *P* = .016). All other races had similar trends in the ECI score compared to White patients which is summarized in Table 3. Trend analysis per procedure is shown in Figure 2A and per race in Figure 2B.

**Table 3:** Slopes for average Elixhauser comorbidity index score and CHA_2_DS_2_-VASc, per year by procedure vs no procedure, race, and comparison of slopes for race with White race as reference group.

	Elixhauser comorbidity index score				CHA_2_DS_2_-VASc			
	Slope	95% lower bound	95% upper bound	*P* value	Slope	95% lower bound	95% upper bound	*P* value
No procedure	0.42	0.40	0.44	<.001	0.051	0.047	0.054	<.001
Endovascular intervention	0.49	0.46	0.53	<.001	0.055	0.049	0.061	<.001
Difference	0.07	0.04	0.11	<.001	0.004	–0.001	0.010	.119
White	0.5	0.48	0.52	<.001	0.06	0.056	0.064	<.001
Black	0.45	0.41	0.49	<.001	0.055	0.048	0.062	<.001
Hispanic	0.46	0.40	0.51	<.001	0.045	0.036	0.053	<.001
AAPI	0.52	0.44	0.60	<.001	0.041	0.027	0.055	<.001
Native American	0.48	0.33	0.63	<.001	0.059	0.029	0.089	<.001
Other	0.49	0.41	0.57	<.001	0.055	0.041	0.069	<.001
vs White	base				base			
Black	–0.05	–0.08	–0.01	.016	–0.005	–0.012	0.002	.173
Hispanic	–0.04	–0.09	0.01	.136	–0.015	–0.024	–0.007	<.001
AAPI	0.02	–0.07	0.10	.668	–0.018	–0.033	–0.003	.016
Native American	–0.02	–0.17	0.13	.811	–0.001	–0.032	0.03	.954
Other	–0.01	–0.09	0.07	.800	–0.005	–0.019	0.009	.484

AAPI, American and Pacific Islander.

**Figure 3:**
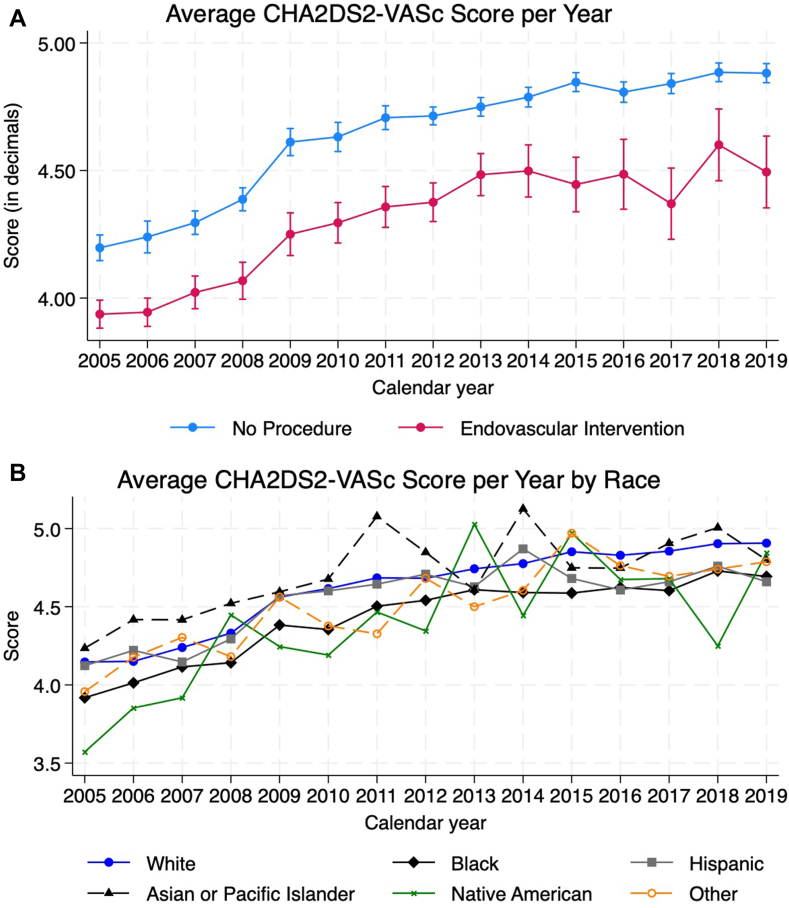
**(A) Average CHA_2_DS_2_-VASc score per year, (B) Average CHA_2_DS_2_-VASc score per year by race**.

The CHA_2_DS_2_-VASc score increased every year by an average of 0.05 per year among those who did not receive an intervention (95% CI, 0.047-0.054; *P* < .001) and 0.06 among those who received an endovascular intervention (95% CI, 0.05-0.06; *P* < .001). There was no difference in the slopes between the 2 groups (difference: 0.004; 95% CI, –0.001 to 0.010; *P* = .12). Compared to White patients, the average CHA_2_DS_2_-VASc score was lower by an average of 0.02 per year for Hispanic patients (95% CI, –0.02 to –0.01; *P* < .001) and lower by 0.02 for AAPI patients (95% CI, –0.03 to –0.003; *P* = .016). All other races had similar trends in the CHA_2_DS_2_-VASc score compared to White patients as shown in Table 3. Figure 3A and B demonstrate the trend analysis per procedure and per race, respectively.

### Multivariable regression

Admissions involving Black patients had lower odds of receiving an endovascular intervention (OR, 0.80; 95% CI, 0.75-0.87; *P* < .001). Admissions involving Hispanic, AAPI, Native American or Other race patients had similar odds of receiving an endovascular intervention compared to White patients (Hispanic: OR, 1.07; 95% CI, 0.97-1.19; *P* = .18; AAPI: OR, 0.95; 95% CI, 0.82-1.10; *P* = .48, Native American: OR, 1.30; 95% CI, 0.89-1.89; *P* = .17 and other: OR, 1.01; 95% CI, 0.87-1.17; *P* = .92, respectively). CKD stage 3 or higher was associated with lower odds of receiving an endovascular intervention (OR, 0.76; 95% CI, 0.72-0.81; *P* < .001). Renovascular hypertension was associated with higher odds of receiving an endovascular intervention (OR, 2.58; 95% CI, 2.41-2.76; *P* < .001).

Endovascular intervention was associated with higher odds of routine discharge (OR, 2.12; 95% CI, 2.02-2.23; *P* < .001). Compared to White patients, Black patients had lower odds of routine discharge (OR, 0.86; 95% CI, 0.82-0.90; *P* < .001). Admissions involving Hispanic, AAPI, Native American or other race patients had similar odds of routine discharge compared to White patients (Hispanic: OR, 1.04; 95% CI, 0.97-1.11; *P* = .28; AAPI: OR, 1.06; 95% CI, 0.96-1.18; *P* = .23, Native American: OR, 1.17; 95% CI, 0.96-1.42; *P* = .12 and other: OR, 1.00; 95% CI, 0.91-1.09; *P* = .95, respectively).

Endovascular intervention was associated with lower odds of in-hospital mortality (OR, 0.63; 95% CI, 0.54-0.72; *P* < .001). Compared to White patients, Black patients had lower odds of in-hospital mortality (OR, 0.86; 95% CI, 0.76-0.98; *P* = .023). Admissions involving AAPI and Native American patients had higher odds of in-hospital mortality compared to White patients (AAPI: OR, 1.37; 95% CI, 1.08-1.73; *P* = .009, Native American OR: 1.60; 95% CI: 1.04-2.48; *P* = .034, respectively). Hispanic and other race patients had similar odds of in-hospital mortality compared to White patients (Hispanic: OR, 0.92; 95% CI, 0.77-1.09; *P* = .33; other: OR, 1.18; 95% CI, 0.92-1.51; *P* = .19, respectively). Additional results can be seen in Table 4.

**Table 4:** Multivariable regression for patient profile of endovascular intervention group, routine discharge, and in-hospital mortality.

	Endovascular procedure			Routine discharge			Died during hospitalization		
	Odds ratio	95% CI	*P* value	Odds ratio	95% CI	*P* value	Odds ratio	95% CI	*P* value
Endovascular procedure				2.12	2.02-2.23	<.001	0.63	0.54-0.72	<.001
Age in years at admission	1.00	0.996-0.999	<.001	0.96	0.96-0.96	<.001	1.03	1.02-1.03	<.001
Female patients	0.85	0.82-0.88	<.001	0.76	0.75-0.78	<.001	0.93	0.87-1.00	.065
Race									
White	1.00	–	–	1.00	–	–	1.00	–	–
Black	0.80	0.75-0.87	<.001	0.86	0.82-0.90	<.001	0.86	0.76-0.98	.023
Hispanic	1.07	0.97-1.19	.176	1.04	0.97-1.11	.281	0.92	0.77-1.09	.325
Asian/Pacific Islander	0.95	0.82-1.10	.479	1.06	0.96-1.18	.230	1.37	1.08-1.73	.009
Native American	1.30	0.89-1.89	.170	1.17	0.96-1.42	.115	1.60	1.04-2.48	.034
Other	1.01	0.87-1.17	.916	1.00	0.91-1.09	.952	1.18	0.92-1.51	.187
CKD Stage 3+	0.76	0.72-0.81	<.001	0.94	0.92-0.97	<.001	0.83	0.77-0.90	<.001
Long-term anticoagulant	0.70	0.63-0.78	<.001	1.10	1.05-1.15	<.001	0.65	0.57-0.74	<.001
Long-term antiplatelet/antithrombotics	0.93	0.85-1.01	.099	1.10	1.05-1.16	<.001	0.75	0.63-0.90	.002
Long-term aspirin	0.67	0.62-0.72	<.001	1.17	1.13-1.22	<.001	0.72	0.64-0.81	<.001
Renovascular HTN	2.58	2.41-2.76	<.001	1.07	1.02-1.13	.012	0.96	0.80-1.14	.610
Hypertension, any	0.94	0.89-1.00	.054	1.18	1.13-1.23	<.001	0.50	0.45-0.55	<.001
Primary expected payer									
Medicare	1.00	–	–	1.00	–	–	1.00	–	–
Medicaid	0.67	0.61-0.73	<.001	0.89	0.83-0.95	.001	1.20	0.98-1.48	.085
Private insurance	0.92	0.87-0.97	.003	1.22	1.17-1.27	<.001	1.03	0.91-1.17	.638
Self-pay	0.64	0.55-0.74	<.001	1.41	1.26-1.57	<.001	1.52	1.12-2.06	.007
No charge	0.98	0.72-1.33	.884	1.57	1.13-2.18	.007	1.34	0.56-3.21	.510
Other	0.79	0.69-0.91	.001	0.99	0.88-1.11	.853	2.27	1.74-2.97	<.001
Bed size of hospital									
Small	1.00	–	–	1.00	–	–	–	–	–
Medium	1.09	0.86-1.40	.475	1.01	0.94-1.08	.829	–	–	–
Large	1.38	1.09-1.74	.007	1.05	0.99-1.12	.108	–	–	–
Location/teaching status of hospital									
Rural	1.00	–	–	1.00	–	–	1.00	–	–
Urban nonteaching	1.98	1.61-2.43	<.001	1.10	1.03-1.19	.008	1.06	0.92-1.22	.422
Urban teaching	1.87	1.53-2.30	<.001	1.09	1.02-1.17	.014	1.18	1.03-1.35	.019
Region of hospital									
Northeast	–	–	–	1.00	–	–	1.00	–	–
Midwest	–	–	–	1.41	1.32-1.51	<.001	1.12	0.99-1.26	.064
South	–	–	–	1.55	1.46-1.65	<.001	1.10	0.98-1.22	.092
West	–	–	–	1.65	1.53-1.78	<.001	1.18	1.03-1.34	.017
Median household income national quartile for patient ZIP code									
0-25 percentile	–	–	–	1.00	–	–	–	–	–
26-50 percentile	–	–	–	1.01	0.97-1.05	.609	–	–	–
51-75 percentile	–	–	–	0.98	0.94-1.02	.305	–	–	–
>75 percentile	–	–	–	0.97	0.92-1.01	.176	–	–	–
Elixhauser scaled score (van Walraven algorithm, mortality)	0.94	0.93-0.94	<.001	0.94	0.941-0.949	<.001	1.10	1.100-1.109	<.001

### Marginal effect analysis: Endovascular intervention on routine discharge by race

Among White patients, endovascular intervention was associated with an increase in the predicted probability of routine discharge by 15.4% (95% CI, 14.5 to 16.2; *P* < .001). For Black patients, it was associated with an increase of 8.8% (95% CI, 6.4 to 11.3; *P* < .001), for Hispanic patients by 12.0% (95% CI, 8.8-15.3; *P* < .001), for Asian/Pacific Islanders by 12.7% (95% CI, 8.0-17.4; *P* < .001), and for the other race category by 15.8% (95% CI, 11.1-20.5; *P* < .001). Among Native Americans, there was no statistically significant change in predicted probability of routine discharge (Coef., 3.1%; 95% CI, –4.5 to 10.6; *P* = .422).

Compared to White patients, the marginal effect of the endovascular intervention on routine discharge was significantly lower for Black patients by 37.5% (95% CI, –50.3 to –24.7; *P* < .001) and lower for Native American patients by 64.5% (95% CI, –103.6 to –25.5; *P* = .001). No significant differences were observed for Asian/Pacific Islanders (Coef., –13.9%, 95% CI, –40.8 to 12.9; *P* = .31), for Hispanic patients (Coef., –18.1% 95% CI, –36.9 to 0.7; *P* = .059) or for the other race category (Coef., 2.50%, 95% CI, –25.0 to 30.1; *P* = .86). These results are also shown in Supplemental Table S2 and Supplemental Figure S1.

### Marginal effect analysis: Selected comorbidities on odds of receiving endovascular intervention by race

Compared to White patients, there was no statistical significance in the marginal effect of obesity (Black: *P* = .98, Hispanic: *P* = .35, Asian/Pacific Islander: *P* = .43, Native American: *P* = .56, other: *P* = .98), CKD stage 3+ (Black: *P* = .51, Hispanic: *P* = .19, Asian/Pacific Islander: *P* = .77, Native American: *P* = .46, other: *P* = .73), and complicated diabetes (Black: *P* = .50, Hispanic: *P* = .47, Asian/Pacific Islander: *P* = .35, Native American: *P* = .96, other: *P* = .93) on the odds of receiving an endovascular intervention by each race.

## Discussion

Among 792,108 admissions involving RAS, our analysis demonstrated that the trends in the proportion of endovascular intervention decreased over the 15 years among all races; the decrease in trends is larger among White patients (Central Illustration). Additionally, patients presenting to hospitals with RAS have more risk factors and are clinically more ill over the years analyzed, with a higher increase among endovascular interventions. Endovascular intervention was associated with higher odds of routine discharge and lower odds of mortality. Additionally, Black patients had lower odds of undergoing endovascular treatment, routine discharge, and in-hospital mortality compared to White patients. Renovascular hypertension was associated with higher odds of endovascular intervention. These results were adjusted by patient demographics, hospital characteristics and ECI, a summative score for comorbid conditions.

**Central Illustration:**
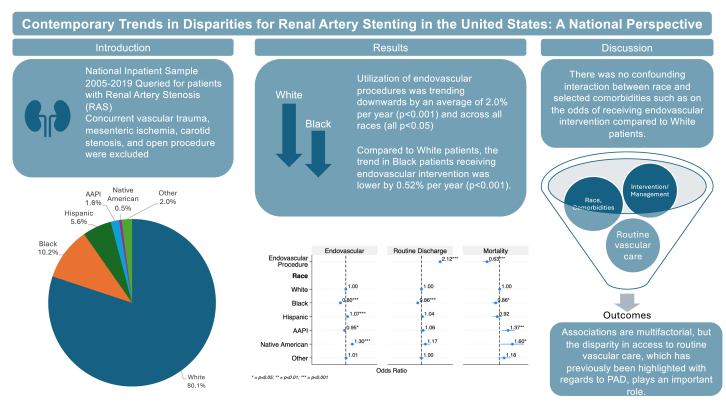
Summary of the study, obtained from the National Inpatient Sample, showing the proportion of races in the cohort. Outcomes shown include the trend analysis, multivariable regression for patient profiles receiving endovascular intervention, routine discharge, and in-hospital mortality. AAPI, American and Pacific Islander.

The 3 major trials, The Cardiovascular Outcomes in Renal Atherosclerotic Lesions (CORAL), Angioplasty and Stenting for Renal Artery Lesions, and Stent Placement in Patients with Atherosclerotic Renal Artery Stenosis and Impaired Renal Function, demonstrated that endovascular intervention plus medical management was not superior to medical management alone for RAS with moderate stenosis.^4^, ^5^, ^6^ This partly accounts for the downward trend in the utilization of endovascular intervention. However, these trials did not specifically analyze patients with resistant hypertension or severe RAS. For example, the Angioplasty and Stenting for Renal Artery Lesions trial only included patients whose physician was uncertain whether they would benefit from revascularization.^6^ The CORAL trial excluded patients with signs of critical RAS, and other trials had exclusion criteria of patients likely to benefit from revascularization.^22^ Meanwhile, the HERCULES trial included patients with refractory hypertension and demonstrated that with an appropriate selection of patients, endovascular intervention significantly reduces systolic blood pressure with better clinical response than medical management.^23^ These findings, along with potentially improved hemodynamics and resolution of symptoms, may also relate to higher odds of routine discharge among patients who received endovascular intervention in the current study.

Although the clinical trials on renal artery intervention have been criticized for their inclusion and exclusion criteria, there was an overwhelming lack of race data provided in these trials. There are 9 randomized controlled trials in the literature regarding percutaneous intervention for renal artery stenting, and only the CORAL trial provided racial data.^4^, ^5^, ^6^^,^^24^, ^25^, ^26^, ^27^, ^28^, ^29^ Although previous literature has shown that race was not a significant predictor of RAS development,^30^ there is a disparity in intervention between the races analyzed in this study. In the present study, Black patients had a lower likelihood of receiving endovascular therapy compared to White patients. However, the proportion of utilization of endovascular intervention is decreasing at a slower rate among Black patients compared to White patients. Meanwhile, the proportion of utilization is decreasing at a similar rate for patients among other races/ethnicities, including Hispanic, Native American, and AAPI, compared to White patients. Black patients also had a lower likelihood of discharge to home. We also demonstrated in our analysis that there was no confounding interaction between race and selected comorbidities such as obesity, CKD stage 3+, and complicated diabetes on the odds of receiving endovascular intervention compared to White patients. Therefore, we may suggest that the difference in selected comorbidities at presentation between race/ethnicity, including those of the Hispanic ethnicity, Native Americans, and AAPI, may not fully explain the disparity in receiving endovascular intervention. These associations are multifactorial, but the disparity in access to routine vascular care, which has previously been highlighted with regard to peripheral artery disease, plays an important role.^31^ Soden et al^31^ demonstrated that Black patients were less likely to receive statin or any antiplatelet prior to and upon discharge from an abdominal aortic aneurysm or peripheral artery disease intervention compared to White patients.

Our study found that the average ECI score among patients with RAS consistently increased over the years analyzed indicating the RAS patient population is burdened by increasing comorbidities and faces the possibility of poorer outcomes as a result. Black patients with RAS have been found to have greater rates of comorbidities including refractory hypertension, coronary artery disease, and cerebrovascular disease compared to White patients, which corroborates with our data.^32^ Additionally, although patients who did not receive intervention had higher average ECI scores baseline, the scores among patients who underwent revascularization increased at a steeper rate, which indicates accelerating comorbidities in this cohort.

### Limitations

This study has limitations. First, the NIS does not capture follow-up data. Thus, long-term outcomes could not be analyzed. Second, each observation represents an admission, rather than a patient. Therefore, some hospitalizations could have been for the same patient requiring repeat procedures. Third, lesion characteristics such as the degree of stenosis were unavailable. However, patients included in this study are hospitalized and therefore may have a moderate-to-severe form of stenosis. Lastly, administrative databases may misclassify diagnoses and procedure codes. This study’s method of selecting admissions was to capture all patients with RAS, but some admissions that involved patients with controlled disease may have been captured.

## Conclusion

The trend in the proportion of endovascular intervention for RAS decreased over the 15 years analyzed for White and non-White patients. However, the decrease in trend was larger among White patients. Additionally, Black patients had lower odds of undergoing endovascular treatment, lower odds of routine discharge, and lower odds of mortality compared to White patients.
