# Supplementary material for: Contemporary Trends in Disparities for Renal Artery Stenting and Angioplasty in the United States
Source: J Soc Cardiovasc Angiogr Interv. 2025 Sep 30;5(3 Suppl):102610. doi: 10.1016/j.jscai.2025.102610 (PMC13112819; doi:10.1016/j.jscai.2025.102610)
Supplement: Supplemental Tables and Figure [file mmc1.docx]

Supplementary Table 1: ICD codes used in this study

| **Procedure/Diagnosis** | **ICD-9** | **ICD-10** |
| --- | --- | --- |
| Renovascular Hypertension | 405.x1 | I50.1 |
| Fibromuscular Dysplasia | 447.3 | I77.3 |
| Renal Artery Stenosis | 440.1 | I70.1 |
| Endarterectomy (Open Procedure) | 38.16 | 04C90ZZ, 04CA0ZZ |
| Open Resection | 38.36, 38.46 | 04B90ZZ, 04BA0ZZ, 04R907Z, 04R90JZ, 04R90KZ, 04RA07Z, 04RA0JZ, 04RA0KZ |
| Open Bypass | 39.24 | 0410093, 0410094, 0410095, 04100A3, 04100A4, 04100A5, 04100J3, 04100J4, 04100J5, 04100K3, 04100K4, 04100K5, 04100Z3, 04100Z4, 04100Z5 |
| Open Reimplantation | 39.55 | 04S90ZZ, 04SA0ZZ, 06S90ZZ, 06SB0ZZ |
| Endovascular Angioplasty | 39.50 | 04793Z1, 04793ZZ, 047A3Z1, 047A3ZZ |
| Endovascular  Stent | 39.90 | 04793[4-7 or D-G], 047A3[4-7 or D-G] |
| CKD Stage 3+ | 585.3, 585.4, 585.5, 585.6 | N18.3, N18.4, N18.5, N18.6 |
